# A cross-sectional study of the magnitude, barriers, and outcomes of HIV status disclosure among women participating in a perinatal HIV transmission study, “the Nevirapine Repeat Pregnancy study”

**DOI:** 10.1186/s12889-015-2345-6

**Published:** 2015-09-29

**Authors:** Flavia M Kiweewa, Paul M Bakaki, Michelle S McConnell, Maria Musisi, Constance Namirembe, Frances Nakayiwa, Fiona Kusasira, Dorothy Nakintu, Michael C Mubiru, Philippa Musoke, Mary Glenn Fowler

**Affiliations:** Makerere University-Johns Hopkins University (MU-JHU) Research Collaboration, Kampala, Uganda; Case Western Reserve University, Ohio, USA; US Department of Health and Human Services, Centers for Disease Control and Prevention, Hanoi, Vietnam; Department of Epidemiology and Biostatistics, Makerere University School of Public Health, Makerere University College of Health Sciences, Kampala, Uganda; Department of Pediatrics and Child Health, Makerere University School of Medicine, University College of Health Sciences, Makerere, Uganda; Johns Hopkins University School of Medicine, Baltimore, MD USA; Makerere University - Johns Hopkins University Research Collaboration, MU-JHU Research Building, Old Mulago Hill Road, P.O. Box 23491 Kampala, Uganda

**Keywords:** HIV status, Disclosure, Perinatal, Mother-to-child, Partner

## Abstract

**Background:**

HIV status disclosure is a difficult emotional task for HIV-infected persons and may create the opportunity for both social support and rejection. In this study, we evaluated the proportions, patterns, barriers and outcomes of HIV- 1 status disclosure among a group of women in Uganda.

**Methods:**

An exit interview was conducted one year post-partum for 85 HIV-infected women who participated in a study of HIV-1 transmission rates among NVP-experienced compared with NVP-naïve women in “The Nevirapine Repeat Pregnancy (NVP-RP) Study” at the Makerere University-Johns Hopkins University Research Collaboration, Kampala-Uganda, between June 2004 and June 2006.

**Results:**

Of the 85 women interviewed, 99 % had disclosed their HIV status to at least one other person. Disclosure proportions ranged between 1 % to employer(s) and 69 % to a relative other than a parent. Only 38 % of the women had disclosed to their sex partners. Women with an HIV-infected baby were more likely than those with an uninfected baby to disclose to their sex partner, OR 4.9 (95 % CI, 2.0 –11.2), and women were less likely to disclose to a partner if they had previously disclosed to another relative than if they had not, OR 0.19 (95 % CI, 0.14–0.52). The most common reasons for non-disclosure included fear of separation from the partner and subsequent loss of financial support 34 %, and not living with the partner (not having opportunities to disclose) 26 %. While most women (67 %) reported getting social support following disclosure, 22 % reported negative outcomes (neglect, separation from their partners, and loss of financial support). Following disclosure of HIV status, 9 % of women reported that their partner (s) decided to have an HIV test.

**Conclusion:**

Results from this study show high overall HIV disclosure proportions and how this disclosure of HIV status can foster social support. However, proportions of disclosure specifically to male sex partners were low, which suggests the need for interventions aimed at increasing male involvement in perinatal care, along with supportive counseling.

## Background

With the introduction of antiretroviral drugs, significant successes have been achieved in the reduction of the risk of HIV transmission from mother to child during pregnancy, delivery and breast feeding in resource-limited settings [1–10]. Despite these successes, significant problems remain that may impact the implementation of HIV mother-to-child transmission (MTCT) interventions in developing countries.

In sub-Saharan Africa, reported proportions of women who disclose their HIV status to sex partners vary widely. Previous studies have found that between 17 % and 86 % of HIV-infected individuals share HIV test results (HIV status) with their sex partners [11–16] with women tested in the context of their antenatal care being less likely to disclose their HIV status to their sex partners than non-pregnant women (16.7 % versus 32 %). In a prevention of mother-to-child transmission (PMTCT) of HIV trial conducted in Tanzania, Kilewo et al. found that only 17 % of the women shared their HIV status with their partner [17]. Such low proportions of disclosure have implications for PMTCT programs as the optimal uptake and adherence to such programs are difficult for such women whose partners are either unaware of or do not understand the benefits of participation in PMTCT programs [18–21].

Research conducted in a variety of settings has shown that there are a number of barriers that HIV-infected individuals face in sharing their HIV test results with friends, family, and, most importantly, sex partners. These barriers include fears of abandonment and loss of economic support, discrimination, accusations of infidelity, violence, upsetting family members, and blame [12, 14, 22, 23]. Despite these fears, disclosure of HIV status to sex partners remains an important public health goal as emphasized by the World Health Organization [24] and the Centers for Disease Control and Prevention [25] in their HIV testing and counseling protocols. In general HIV disclosure is an essential aspect in the prevention, care, treatment and support for HIV-infected persons [26]. In the context of PMTCT, disclosure to male partners has been associated with improved adherence to PMTCT regimens [27], better infant feeding practices [28], safer sex practices, and increased male partner testing [29]. Conversely, HIV-infected women who have not disclosed their HIV-1 status to their partner are more likely than other HIV-infected women to have suboptimal adherence to PMTCT regimens [26, 30, 31], higher drop-out rates from PMTCT programs [32], and fewer infants tested for HIV-1[9]. Given the pivotal role of disclosure in PMTCT, understanding the problems faced by HIV infected women concerning HIV status disclosure is critical in informing future MTCT intervention program activities in resource limited settings.

The aim of this study was to describe the proportions, patterns, barriers and outcomes of HIV status disclosure among HIV-infected women participating in a study of mother-to-child HIV-1 transmission rates among NVP-experienced compared to NVP-naïve women, “The Nevirapine Repeat Pregnancy (NVP-RP) Study”.

## Methods

### The Nevirapine Repeat Pregnancy (NVP-RP) study

The NVP-RP study was an observational study of mother-to-child HIV-1 transmission conducted at Makerere University-Johns Hopkins University (MU-JHU) Research Collaboration, Kampala – Uganda between June 2004 and June 2006, the details of which have been previously published [33]. Briefly, the main objectives of the NVP-RP study were to determine HIV transmission rates in infants born to HIV-infected women who received single dose NVP in a previous pregnancy as well as in the repeat pregnancy (NVP-experienced) and to compare these transmission rates in infants born to HIV-infected women who had not received prior single dose NVP (NVP-naïve). Two groups of HIV-positive women, previously pregnant while in the HIV Network for Prevention (HIVNET) 012 trial[2] or PMTCT Program (retrospective group) or currently pregnant (prospective group), were enrolled in this study. The prospective mother and infant group included women who were pregnant at the time of study enrollment and were either NVP-experienced or NVP-naïve.

Women were recruited between 28 and 40 weeks of gestation from the antenatal clinics of Mulago National Referral Hospital in Kampala and followed for one year. As part of the study, women received counseling and support for HIV status disclosure during the one year follow-up period.

### Procedures

An exit interview was conducted among 85 HIV-1 infected mothers at their 12 month (final) visit in the prospective arm of the NVP-RP study at MU-JHU Research Clinic after obtaining separate informed consent. Data were collected between November 2005 and June 2006. Disclosure was defined as the individual revealing her HIV status to her primary sex partner, parent, sibling, other relatives, employer, or friends, at any time since diagnosis. Proportions of women who had disclosed to a significant other (including a primary sex partner), as well as predictors and outcomes of disclosure were determined.

### Measurements

Data was collected using a pre-tested standardized questionnaire. The primary outcome variable for this study was self-reported HIV status disclosure recorded as ‘yes’or ‘no’. Other outcome variables included disclosure outcomes for example receipt of social support, partner deciding to test, neglect and separation, etc. Explanatory variables included socio-demographic characteristics (age, income, education level, religion, marital status, and occupation), relationship factors (reaction from significant other[s], partner’s HIV status (as reported by the participant), disclosure barriers, and psychosocial factors (stigma, and social support).

Single response multiple choice questions were asked to determine disclosure patterns: ‘Have you ever told any of the following people about your HIV status?’ The outcome of disclosure to the primary sex partner or significant other was then assessed. Data were collected on disclosure outcomes based on responses to the following question: ‘What happened when you disclosed your HIV status?’ For participants who had not disclosed to their sex partner the reasons for non-disclosure were probed. Participants who knew whether their partners had ever tested for HIV were asked whether their partners had tested HIV positive as well ‘Has your partner ever been tested for HIV? If yes, did your partner test positive?’

### Statistical methods

STATA version 13.0 was used for data analyses. All variables were checked for missing data and inconsistencies. Distribution of the data using summary measures and graphical displays were examined to identify outliers and verified against source documentation. Participants were compared for baseline characteristics, disclosure proportions, barriers and outcomes of disclosure using descriptive statistics.

To determine predictors of disclosure a logistic regression model was run with disclosure as the outcome variable. Bivariate analyses were used to determine the presence of statistically significant associations between explanatory variables and the outcome variable. To identify independently associated factors, multiple logistic regression was employed. Factors associated with disclosure at a *p* value of less than 0.1 in the bivariable analysis were subsequently entered into backward stepwise logistic regression models to determine which factors were independently associated with disclosure. A *p*-value of ≤ 0.05 was considered statistically significant in the final model.

### Ethical considerations

The study was approved by institutional review boards at the Uganda Virus Research Institute, Uganda National Council of Science and Technology in Uganda and the Centers for Disease Control and Prevention in Atlanta, Georgia. Written informed consent for study participation was obtained from all participants.

## Results

Table 1 presents the demographic characteristics of the 85 women who participated in this study. There were no missing data or outliers. Women who participated had a mean age of 28.7 years and 50 % had completed primary school. The majority (60 %) were living with their partner in a consensual relationship, and a lower proportion was married (13 %), single (13 %) or had divorced or separated from their spouses at the time of the interview (14 %). Among 66 women who were married or were living in consensual relationships, 50 % were housewives or homemakers, who did not work outside the home. Only 39 % of the 85 women were aware of their spouses or partners ever testing for HIV, and 31 % reported having known HIV positive partners.

**Table 1:** Demographics of participants in the Nevirapine Repeat Pregnancy sub-study on HIV status disclosure, Uganda, 2004–2006

Characteristic	Mean or proportion (n [%]), *N* = 85
Age (years) mean	28.7
Marital status	
Married	11 (13)
Consensual/cohabiting relationship	51 (60)
Single	11 (13)
Separated/divorced	12 (14)
Education	
None	5 (6)
Primary school completed	43 (50)
Secondary school completed	31 (37)
Tertiary school completed	6 (7)
Occupation	
Works from home	33 (39)
Teacher	5 (6)
Farmer	3 (3)
Business	6 (7)
Vendor	23 (27)
Other (waitress, housemaid)	15 (18)
Partner ever tested for HIV	
Yes	33 (39)
No	23 (27)
Do not know	29 (34)
HIV status of partner	
Partner positive	26 (31)
Partner negative	7 (8)
Unknown (partner not known to have tested)	52 (61)
HIV status of the study child	
HIV infected	15 (18)
Not HIV infected	70 (82)

### Disclosure proportions

**Fig. 1:**
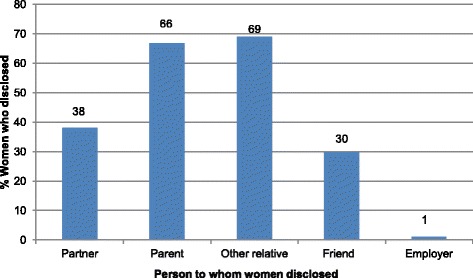
HIV status disclosure among women in the Nevirapine Repeat Pregnancy sub-study Uganda, 2004–2006

Of the 85 women interviewed, 99 % had disclosed their HIV status to at least one other person, and only one woman had never told anyone that she was HIV infected. Of the 84 women who had disclosed their HIV status, 38 % had disclosed to their sex partners, 66 % to their parents, 69 % to other relatives, and 30 % to friends. Only 1 % had disclosed to her employer (Fig. 1).

### Barriers to partner disclosure

Among the 53 women who had not yet disclosed their HIV status to their partners reported barriers to disclosure included fear of separation and subsequent loss of financial support (34 %), separated from or not living with partner (not having opportunities to disclose) (26 %), and stigmatization (2 %). Other reasons, included fear of causing worry to the partner or fear of a harmful reaction from the partner (e.g., partner causing self-harm or becoming abusive) and not yet being ready to disclose (38%) (data not shown).

### Outcomes of disclosure

Outcomes of disclosure included receipt of social support (67%) neglect/separation from partner (8 %), negative reactions (violence, stigmatization, confidants telling others) (9 %), loss of monetary support (5 %), and other reactions, including indifference (especially from partners), partner not believing the woman and friends motivated to go for HIV testing (13 %) (Table 2). Among the 32 women who disclosed their HIV status to their partner, only 25 % reported that their partner subsequently underwent HIV testing as a result of the disclosure the remaining 75 % women reported that their partners had already had an HIV test before the disclosure.

**Table 2:** Outcome of disclosure of participants in the Nevirapine Repeat Pregnancy sub-study, Uganda, 2004-2006

Outcome^a^	n (%) *N* = 85
Received social support	57 (67 %)
Partner decided to test	8 (9 %)
Neglect/separated from partner	7 (8 %)
Loss of monetary support from partner	4 (5 %)
Other negative reactions (violence, stigmatization, confidants telling others)	8 (9 %)
Other outcomes^b^	11 (13 %)

^a^One or more outcomes may be indicated by each participant

^b^“Other” includes; partner indifferent, partner did not believe her, friends did not believe her, sibling advised her not to tell partner, initially separated bed rooms with partner, friends were encouraged to go for HIV testing as well

All women who had disclosed to their partners knew the partner’s HIV status. One woman in a sero-discordant relationship knew her partner’s HIV status but had not disclosed her own status to him.

### Predictors of disclosure to partner

**Fig. 2:**
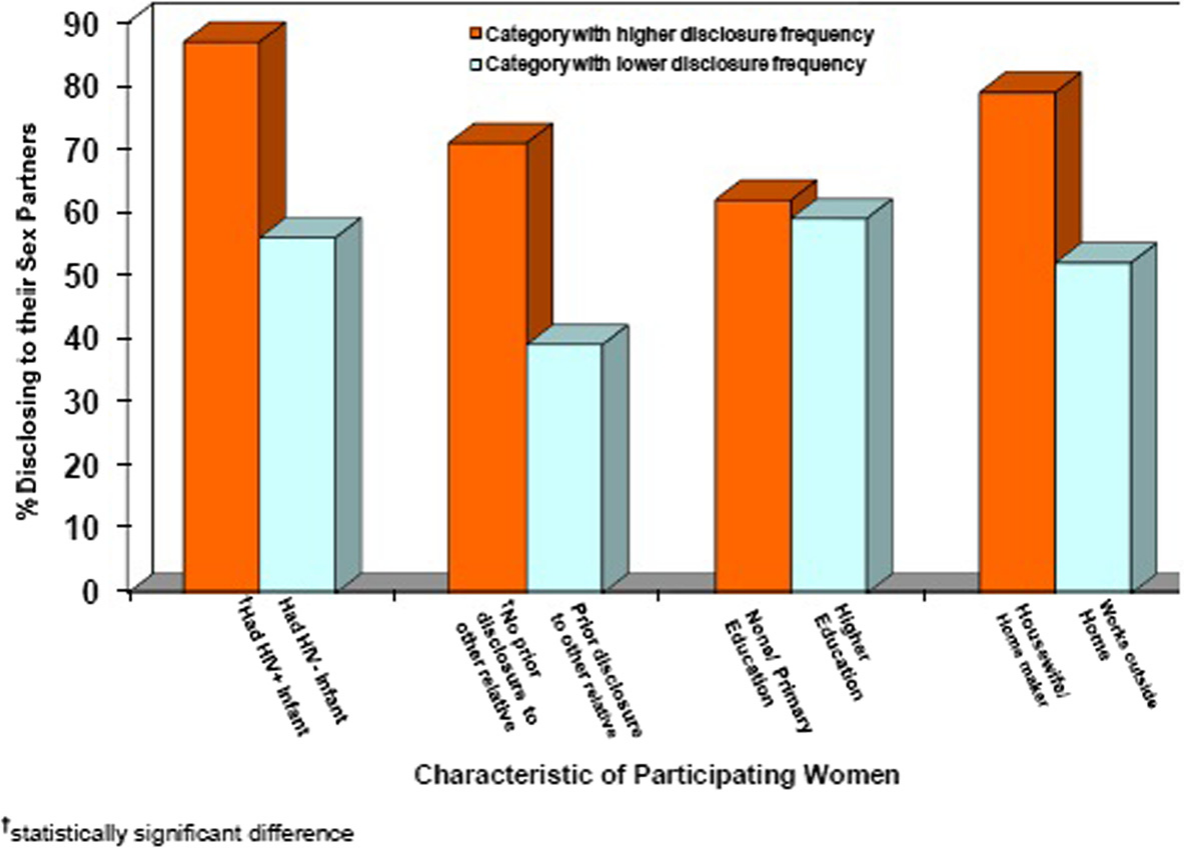
Proportion of women disclosing HIV serostatus to sex partners, by selected characteristic, Nevirapine Repeat Pregnancy sub-study, Uganda, 2004–2006

Figure 2 shows proportions of HIV status disclosure by selected characteristics. In bivariate analyses HIV status disclosure to the sex partner was positively associated with having an HIV-infected study baby, OR 6.7 (95 % CI, 1.4 – 14.2), not having disclosed to a relative (other than a parent), OR 6.1 (95 % CI, 1.7 – 9.2) and being a housewife or homemaker, OR 4.1 (95 % CI, 1.3 – 13.2). After adjusting for age, education level, and marital status, the association remained significant with having an HIV-infected baby (adjusted OR 4.9 [95 % CI, 2.0 – 11.2]) and not having previously disclosed to a relative other than a parent (adjusted OR 0.19 [95 % CI, 0.14 – 0.52]), but not with being a housewife or homemaker.

## Discussion

The general level of disclosure in this study was very high, with 99 % of the women reporting having disclosed to at least one other person. As would be expected, the women disclosed selectively, with the majority disclosing to only one person, most often to a parent (67 %) or a relative other than a parent (69 %).

These findings are comparable to other studies in Africa, with disclosure proportions of greater than 90 % to at least one other person [12, 34–36]. In a study conducted in Uganda, Nakayiwa et al. found that 97 % of HIV infected patients in an HIV care program had ever disclosed their HIV status to at least one other person [34]. Similarly, two other studies in South Africa found that more than 90 % of HIV infected study participants had disclosed their HIV status to one or more person [12, 35].

Despite the high levels of disclosure, in general, the proportion of women disclosing to a sex partner was relatively low in this study, with only 38 % of all women having disclosed to their partners. In addition, most women did not know their partner’s HIVstatus, which is similar to findings from previous studies [15, 37]. The reasons given for non-disclosure to the partner included not being ready to disclose, not having sufficient time or opportunities to disclose (e.g., separation from partner, not having enough time with partner), and fear of negative consequences from the disclosure (e.g., fear of being stigmatized, partner causing self-harm, partner becoming abusive). However, the majority of the women who had disclosed reported getting social support, and some reported that their partners decided to test for HIV following disclosure. Negative outcomes were less common but not rare, and included neglect and/or separation from partner, violence from partner, stigmatization by relatives, confidants disclosing to others, and indifference – especially from partners.

The relatively low proportion of women who disclosed to their sex partner in our study suggests considerable complexities around issues of partner disclosure. Partner factors and relationship dynamics remained the key to understanding why women do or do not disclose their HIV status. Notably, women with an HIV–infected study baby, or who had not previously disclosed to a relative were more likely than other women to disclose to their partners. Women who disclosed to a relative could have been less likely to subsequently disclose to their partners due to the fact that they received the support they felt they needed. Another explanation may be that women who wanted to disclose but were reluctant to tell their partner may have felt that their only alternative was to disclose to a relative. In the same way, in order to get the support needed in the care for an HIV infected child, women with an HIV infected study baby may have found it necessary to disclose to their partners. The relatively low prevalence of disclosure to sex partners observed in this study suggests a potential risk for transmission in cases where the male partner is uninfected or of unknown status. This is particularly so if non-disclosure leads to not using condoms during sex and perhaps placing the partner at risk of HIV infection [38]. These gaps in disclosure to sex partners highlight the need for guidance regarding counseling women on disclosing their HIV status, including for cases in which the woman might be at risk for negative consequences such as violence and loss of housing or of financial support.

Our study had some limitations. First, the parent study was not designed to assess disclosure as a primary outcome; information from women participating in a perinatal research study may have limited generalizability. Participants had been diagnosed over a year before the exit interview and had undergone several counseling sessions as part of the primary study protocol, which encouraged them to disclose to a sex partner or other close friend or relative. This counseling may explain the high proportions of disclosure reported in this study. This finding is consistent with results from a study by Mansergh et al., who found that the length of time since diagnosis was positively correlated with disclosure [39]. Additionally, our study relied on self-report, which made the information subject to social desirability reporting bias. To minimize social desirability bias, women were counseled by trained counselors in a supportive and non-judgmental way.

## Conclusion

 Results from this study show high general HIV disclosure proportions and how this disclosure of HIV status can foster social support. The study findings may also suggest areas for further intervention research. Specifically, intervention models aimed at increasing male involvement in perinatal care, along with providing supportive counseling may be feasible approaches to increasing the proportion of women disclosing their HIV status to their male partners.

## Abbreviations

PMTCT: Prevention of mother-to-child transmission of HIV
HIV: Human immuno deficiency virus
NVP-RP Study: Nevirapine Repeat Pregnancy study
